# Expression of Soluble Forms of Yeast Diacylglycerol Acyltransferase 2 That Integrate a Broad Range of Saturated Fatty Acids in Triacylglycerols

**DOI:** 10.1371/journal.pone.0165431

**Published:** 2016-10-25

**Authors:** Nawel Haïli, Julien Louap, Michel Canonge, Franjo Jagic, Christelle Louis-Mondésir, Thierry Chardot, Pierre Briozzo

**Affiliations:** Institut Jean-Pierre Bourgin, INRA, AgroParisTech, Université Paris-Saclay, Versailles, France; Universidade Federal do Rio Grande do Sul, BRAZIL

## Abstract

The membrane proteins acyl-CoA:diacylglycerol acyltransferases (DGAT) are essential actors for triglycerides (TG) biosynthesis in eukaryotic organisms. Microbial production of TG is of interest for producing biofuel and value-added novel oils. In the oleaginous yeast *Yarrowia lipolytica*, Dga1p enzyme from the DGAT2 family plays a major role in TG biosynthesis. Producing recombinant DGAT enzymes pure and catalytically active is difficult, hampering their detailed functional characterization. In this report, we expressed in *Escherichia coli* and purified two soluble and active forms of *Y*. *lipolytica* Dga1p as fusion proteins: the first one lacking the N-terminal hydrophilic segment (Dga1pΔ19), the second one also devoid of the N-terminal putative transmembrane domain (Dga1pΔ85). Most DGAT assays are performed on membrane fractions or microsomes, using radiolabeled substrates. We implemented a fluorescent assay in order to decipher the substrate specificity of purified Dga1p enzymes. Both enzyme versions prefer acyl-CoA saturated substrates to unsaturated ones. Dga1pΔ85 preferentially uses long-chain saturated substrates. Dga1p activities are inhibited by niacin, a specific DGAT2 inhibitor. The N-terminal transmembrane domain appears important, but not essential, for TG biosynthesis. The soluble and active proteins described here could be useful tools for future functional and structural studies in order to better understand and optimize DGAT enzymes for biotechnological applications.

## Introduction

Triglycerides (TG) are neutral lipids composed of glycerol esterified to three fatty acyl groups. They are the major energy storage molecules in most eukaryotic organisms. They have multiple functions, mainly serving as a reservoir of fatty acids (FA) for energy production and membrane biosynthesis. They can also play a major role in cell growth and development [1]. TG biosynthesis can occur through multiple pathways, including acyl-CoA independent ones [2]. However, the major pathway conserved in eukaryotes is known as the Kennedy pathway, or glycerol-3-phosphate pathway, using specific acyltransferases [3]. The final and the only committed step of this TG biosynthesis pathway [1] is the linkage of a sn-1,2-diacylglycerol (DG) to an acyl-CoA, a reaction catalyzed by acyl-CoA:diacylglycerol acyltransferases (DGAT). DGAT enzymes exist as two main families of integral membrane proteins, namely DGAT1 [4] and DGAT2 [5]. These families do not share significant sequence similarity and differ in their biochemical, cellular and physiological roles [1]. They are encountered in animals, plants and yeasts. DGAT1 enzymes belong to the superfamily of membrane bound O-acyltransferases [6]. DGAT2 enzymes are members of the acyl-CoA:monoacylglycerol acyltransferase gene family [1]. Beside membrane-bound DGAT enzymes, cytosolic proteins possessing acyltransferase activity have been described in plants. They belong to a new and smaller family of DGAT3 enzymes [7], but the characterization of their precise catalytic function remains preliminary.

DGAT enzymes are of great importance for the accumulation of TG in animals, plant seeds, and yeasts. As the excessive accumulation of TG favors human diseases such as obesity, liver steatosis and type 2 diabetes, inhibition of DGAT activity is of interest for the control of fat storage in a therapeutical context [8]. Conversely, an increased DGAT activity may permit higher accumulation of oil in crops or in yeasts for biotechnological applications [9, 10]. Transesterification of TG can produce biofuel, and tailoring FA composition in TG can be useful for developing specific industrial products such as lubricants, surfactants, antifoams or biopolymers [11]. In nature, some microorganisms, including bacteria, algae and yeasts, have a great propensity to accumulate substantial amounts of oil. Microorganisms that are naturally capable to synthesize and accumulate oil so that lipids represent at least 20% of their cellular dry weight (CDW) are called oleaginous [9, 12]. Among them, the oleaginous yeast *Yarrowia lipolytica* has an excellent lipid accumulation capacity, mainly under the form of TG [13], that can exceed 50% of CDW [14], and 80% of CDW under genetic modifications [15]. It is a non-pathogenic ascomycetous yeast often found in lipid-rich environments such as dairy products and oily waste. Its ability to grow in hydrophobic substrates makes it an important biotechnological tool. Upon engineering, this yeast can produce and store various fatty acids (ricinoleic acid, conjugated linoleic acid or polyunsaturated fatty acids) or derived products such as dicarboxylic acids. *Y*. *lipolytica* is of interest for applications in fields as different as nutraceuticals, green chemistry or energy [16]. Reverse genetic studies demonstrated that the *Y*. *lipolytica DGA1* gene, a member of the DGAT2 family that encodes the Dga1p enzyme, plays a major role in TG biosynthesis [17]. The same is true for the Dga1p ortholog in *Saccharomyces cerevisiae* [18], which is responsible for 87% of DG esterification activity *in vivo* [19]. Studies in mice and rat cells in culture have provided evidence that DGAT2, more so than DGAT1, is responsible for the majority of TG production [20].

DGAT1 and DGAT2 enzymes contain more than 40% of hydrophobic residues. They are integral membrane proteins with several transmembrane (TM) domains. Their heterologous expression and purification proved to be very difficult [21]. Therefore, very little is known regarding their three-dimensional structure. A main objective of this study was to produce more soluble versions of DGATs in order to analyze their functional and structural properties. DGAT1 enzymes are predicted to contain eight to ten TM domains [7], and can form homotetramers [22]. They are localized in the endoplasmic reticulum, where enriched DGAT activity is found and TG synthesis occurs [7]. DGAT2 enzymes contain only two predicted N-terminal TM domains and are monomeric. In addition to their insertion in the endoplasmic reticulum, they are present and active in lipid droplets [23], [24], in accordance with their important implication in TG biosynthesis. DGAT2 enzymes therefore appear better suited for expression under soluble and active form. Recently, DGAT2 from peanut [25] and from tung tree [26] has been expressed in *Escherichia coli*, but no *in vitro* activity of the purified proteins was reported. In addition, whereas there is a wealth of published data about DGAT2 importance in lipids accumulation [17], results on purified DGAT2 activity and specificity remain scarce. In this work, we expressed in *E*. *coli* two soluble forms of the *Y*. *lipolytica* Dga1p enzyme (fused to the maltose-binding protein (MBP) tag): the first recombinant enzyme lacked 19 N-terminal residues, predicted to be disordered by Phyre2 [27]; the second one lacked the 85 N-terminal residues that include a predicted TM domain. Then, with an *in vitro* fluorescent assay, we showed that the purified enzymes are active and stable. This allowed us to study their substrate specificity, and their sensitivity to a specific DGAT2 inhibitor.

## Materials and Methods

### Transmembrane domains prediction

The TOPPRED 1.10 membrane proteins topology prediction program [28] was used, with default parameters: GES (Goldman-Engelman-Steitz) hydrophobicity scale, certain and putative cuttofs 1.0 and 0.6, respectively. Hydrophobicity was calculated using a core window size of 11 and a full window of 21 amino-acids. The Constrained Consensus TOPology (CCTOP) server [29], which utilizes ten different topology prediction methods, was used in parallel to validate the results.

### Plasmids construction and expression in *E*. *coli*

**Fig 1:**
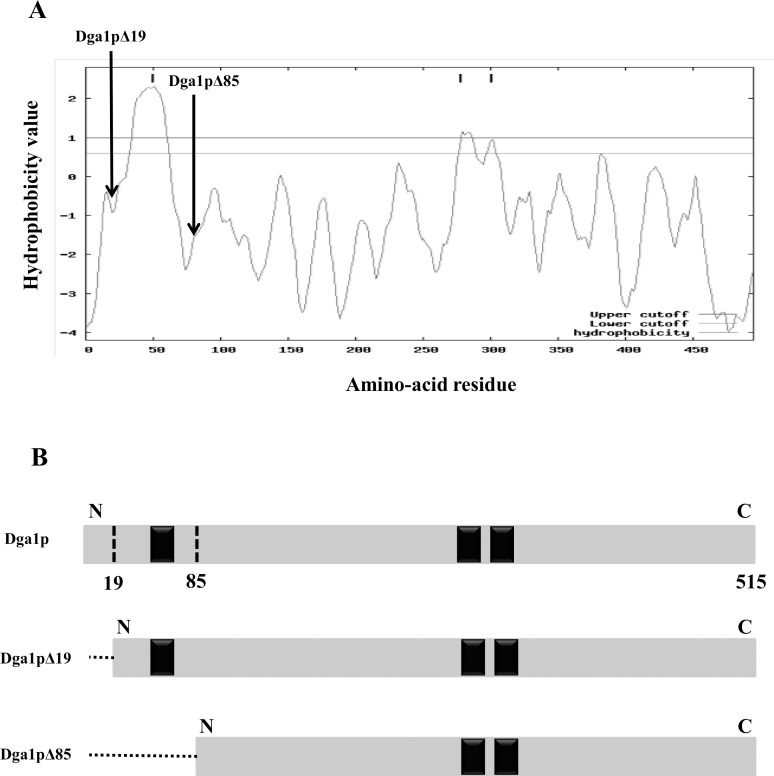
Hydropathy plot analysis and design of Dga1p cloned forms. A: The TOPPRED 1.10 topology prediction program [46] identified a large hydrophobic region at the N-terminus of Dga1p. The cutoff for certain (*upper cutoff = 1*) and for putative (*lower cutoff = 0*.*60*) TM segments are represented. Thick bars indicate the putative integral membrane domains. Thick arrows represent the starting residue of each Dga1p form cloned. B: Schematic representation of Dga1p proteins designed for soluble expression in *E*.*coli*. They were cloned into the pMalc2x vector, without the disordered region (19 amino acids) predicted by Phyre2 for Dga1pΔ19, and without the TM domain (85 amino acids) predicted by TOPPRED 1.10 for Dga1pΔ85. Black rectangles represent the putative TM domains.

PCR was applied on *Y*. *lipolytica* genomic DNA (generous gift of Dr AM Krutz) using Phusion High-Fidelity DNA polymerase (New England Biolabs, Evry, France), forward (Dga1pΔ19: CGC**GGATCC**CACATGGCGGGAATCCGATATG, Dga1pΔ85: CGC**GGATCC**CACATGGTCAAGCGATACTCG) and reverse (DGA1rev: GCG**TCTAGA**CCTAGGTTACTCAATCATTCGGAACTCTGGG) primers. These PCR primers harbor *BamHI* and *XbaI* restriction endonuclease recognition sites (in bold), respectively. They were designed to amplify two constructs: Dga1p lacking the first 57 bp (that encode the 19 N-terminal residues) in order to produce Dga1pΔ19 protein, and Dga1p lacking the first 255 bp (encoding the 85 N-terminal residues) to produce Dga1pΔ85 protein (Fig 1). PCR products were cloned into the pMalc2X vector permitting the expression of the MBP fused at the N-terminus. The cloned vector was transformed into chemically competent *E*. *coli* BL21 (DE3) pLysS strain, using heat shock at 42°C. Transformed bacteria were grown overnight at 37°C on Luria-Bertani (LB) agar plates supplemented with 100 μg/ml ampicillin. Colonies were tested for the presence of the insert by PCR and subsequent sequencing (Beckman Coulter Genomics, Takeley, UK). Bacteria were stored in glycerol stock (50% v/v) at -80°C. *E*. *coli* cells containing pMalc2X vector (control strain), recombinant pMalc2X-Dga1pΔ19 or pMalc2X-Dga1pΔ85 were cultured in LB medium supplemented with 100 μg/ml ampicillin and 20 μg/ml chloramphenicol up to ~0.6 A_600nm_. Cultures were then induced by adding 1 mM isopropyl β-D-1-thiogalactopyranoside (IPTG), after which bacteria were grown overnight under shaking, at room temperature in order to minimize accumulation of non-soluble forms of the protein in inclusion bodies. They were harvested by centrifugation and frozen at -20°C until use. The frozen pellets were resuspended at 4°C in lysis buffer (50 mM Tris–HCl pH 7.5, 150 mM NaCl, 5% (v/v) glycerol, 1 mM EDTA and 1 mM *tris*(2-carboxyethyl)phosphine), and disrupted using a cell disrupter (Constant Systems Ltd, Daventry, UK) operated at 1.96 kbar, then centrifuged at 12,000× *g* for 20 min at 4°C. The membranes and soluble proteins recovered in pellet and supernatant phases, respectively, were analyzed on 10% NuPAGE Bis-Tris gels, using lithium dodecyl sulfate sample buffer (Invitrogen, Cergy-Pontoise, France). Samples were denatured 4 min at 95°C before loading. Proteins were stained with Coomassie Brilliant Blue R-250.

### Purification of recombinant Dga1p constructs

All purification steps were performed at 4°C. The extracted soluble proteins fused to MBP were applied onto an amylose resin (New England Biolabs) pre-equilibrated with lysis buffer. The bound proteins were eluted with the same buffer supplemented with 10 mM maltose, and 0.2% (v/v) glucose. Protein fractions were analyzed on 10% Nu-PAGE, and the pure fractions were pooled. Absorbance spectroscopy at a wavelength of 280 nm was used to determine the protein concentration using the calculated theoretical extinction coefficient. Purified fractions were incubated overnight with lysis buffer supplemented with 0.5% (w/v) N-lauroyl sarcosine, then purified onto a size-exclusion gel chromatography column (Hiload™ 16/60 Superdex™ 200 sephacryl, GE Healthcare, Vélizy, France). The absorbance was monitored at 280 nm, and 2 mL fractions were collected and analyzed on 10% Nu-PAGE.

### Enzymatic assays on recombinant Dga1p constructs

#### Lipids detection assay

DGAT assays were performed according to [30] with modifications. 400 μM of 1,2-dioleoyl-*sn*-glycerol (Cayman Chemical, Ann Arbor, MI) was suspended by vortexing in 0.1 M Tris-HCl buffer pH 7 containing 20% (v/v) glycerol. 50 μg of enzyme purified by amylose affinity chromatography, 400 μM of stearoyl-CoA (C18:0-CoA), 20 mM of MgCl_2_ and 0.01% (v/v) tween 20 were then added. This reaction mixture (100 μl) was incubated 1 hour at 31°C. The reaction was stopped by adding 1 ml of chloroform/methanol (2:1 v/v) mixture and 100 μl of water. After vortexing and centrifugation, the organic phase was evaporated under nitrogen gas. Lipids were then resuspended in 200 μl of chloroform/methanol (2:1 v/v). They were separated on high performance thin layer chromatography (HPTLC) system (CAMAG, Muttenz, Switzerland) in hexane/diethyl ether/acetic acid (80/20/2), using silica-coated aluminum plates (HPTLC Silica gel 60, size 20 x 10 cm, Merck, Darmstadt, Germany). Lipids were visualized after plate staining in 10% (w/v) CuSO_4_, 4% (v/v) phosphoric acid, and 4% (v/v) sulfuric acid in methanol, followed by 30 min drying at 140°C. We used a mixture of markers from Sigma-Aldrich (Saint-Quentin Fallavier, France) including fatty acid (oleic acid), fatty acid methyl ester (oleic acid methyl ester), triglyceride (trioleine), sterol (cholesterol) and sterol ester (cholesteryl oleate) as references (2.7 μg each loaded).

#### Fluorescent assay

The DGAT activity assay used was based on [31], with several modifications. We used 1-{N-[(7-nitro-2-1,3-benzoxadiazol-4-yl)-methyl]amino-decanoyl-2-decanoyl-*sn*-Glycerol (NBD-DG, Cayman chemical) as a fluorescent substrate. 8 μg of the amylose affinity purified enzymes were added to a reaction mixture containing 0.1M Hepes buffer pH 7.6, 0.5 mM ATP, 0.01% (v/v) tween 20, 20 mM MgCl_2_, 0.62 mg/ml BSA and 100 μM NBD-DG in a final volume of 50 μl. In order to investigate enzyme specificity, the reaction mixture was incubated with various fatty acyl-CoAs (lithium salts from Sigma-Aldrich, used at concentrations from 0 to 100 μM): lauroyl-CoA (C12:0-CoA), palmitoyl-CoA (C16:0-CoA), stearoyl-CoA (C18:0-CoA), oleoyl-CoA (C18:1-CoA), and linoleoyl-CoA (C18:2-CoA). The reaction was carried out at 31°C for 1 hour under shaking, then stopped by the addition of chloroform/methanol (2/1 v/v). Lipids were extracted using Folch method [32], dried under nitrogen and resuspended in 200 μl of chloroform/methanol (2/1 v/v) before separation on silica-coated aluminum plates. Lipids were separated on HPTLC in diethyl ether/hexane/methanol/acetic acid (60/40/5/1). The developed TLC plates were then visualized using a Typhoon multi-purpose imager (GE Healthcare). Fluorescent products were quantified using Multi Gauge software, version 3.0 (Fujifilm, Tokyo, Japan). DGAT activity was expressed as picomoles of TG formed per minute per milligram of purified protein, using a calibration curve of the NBD-DG substrate. The DGAT inhibitor niacin used for some experiments was from Sigma-Aldrich.

## Results

### Overexpression and purification of soluble forms of Dga1p from *Yarrowia lipolytica*

**Fig 2:**
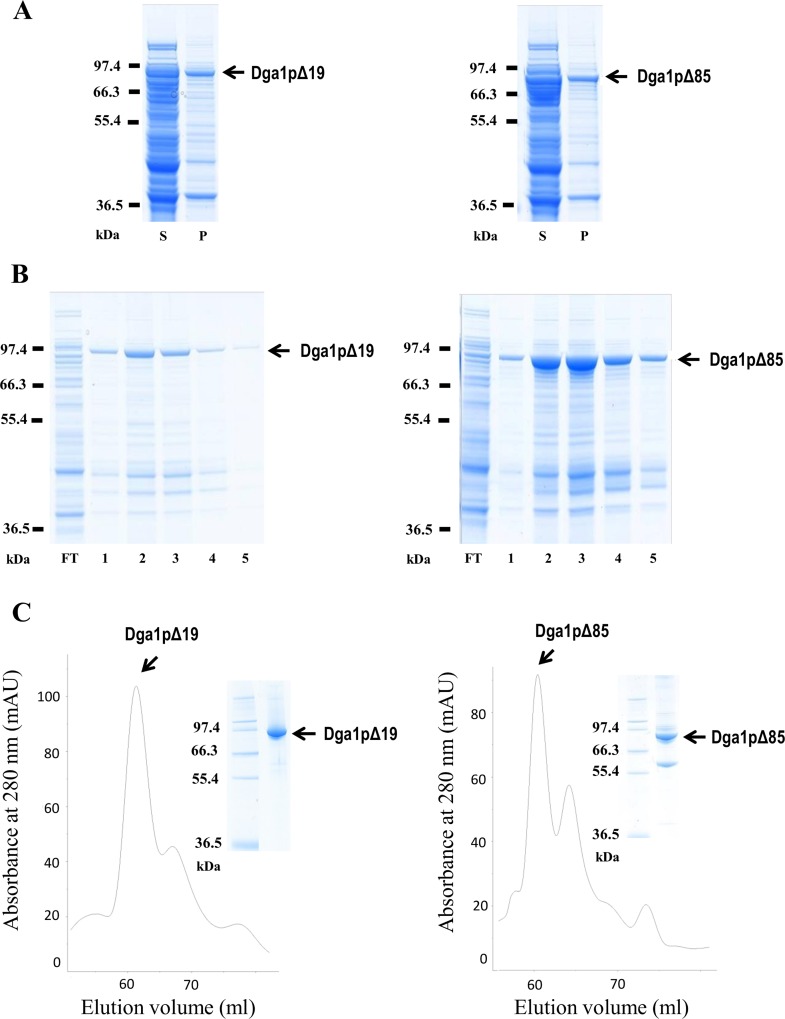
Soluble expression and purification of the two Dga1p constructs. A: Nu-PAGE pattern of recombinant MBP fusion proteins Dga1pΔ19 and Dga1pΔ85 expressed in *E*. *coli*. *S*: soluble fraction of cell lysate, *P*: Pellet (insoluble fraction). Protein marker sizes are shown on the *left* hand side of the gels. B: Affinity purification of MBP-Dga1p proteins from soluble fraction of cell lysate. A total of 2 μg (*left*) and 8 μg (*right*) of proteins were loaded per well. *FT*: flow-through, *1* to *5*: successive eluted fractions. C: Size-exclusion chromatography. Inlet shows on the *right* lane the purity of the fraction from the highest peak as assessed by PAGE, with molecular weight markers on the *left* lane.

The oleaginous yeast *Y*. *lipolytica* contains two DGAT enzymes; the major TG synthesizing enzyme is Dga1p which belongs to the DGAT2 family. Dga1p is encoded by the YALI0E32769g gene (GenBank accession number NC_006071) which codes a 515-residues protein with a molecular mass of 57.8 kDa. Hydropathy plot on Dga1p amino-acid sequence was generated by TOPPRED 1.10. The resulting topology prediction indicates three putative transmembrane domains (Fig 1A). The first one, the higher score of which indicates a strong probability of existence, is located in the N-terminal part of the protein (residues 51–71), and the two other ones, which hydrophobicity values are significantly lower, correspond to residues 279–299 and 302–322. In order to further confirm this conjecture, we used the membrane topology prediction web server CCTOP. All ten programs predicted a TM domain in the 51–71 region, whereas five of them did not predict any in the 279–322 region. Numerous attempts to express the full-length enzyme harboring either 6His or MBP tags were unsuccessful. Using either the pMalc2X or the pDEST17 vectors, and different IPTG induction conditions (37°C, room temperature, 16°C or 4°C, with durations from 3 hours to 4 days), we never obtained a satisfying expression in terms of amount or solubility. Therefore, in order to produce a soluble form of the Dga1p protein, we expressed in *E*. *coli* a Dga1p fragment, named Dga1pΔ85, lacking the 85 N-terminal residues predicted to contain a trans-membrane domain (Fig 1B). We also produced a Dga1p fragment closer to the wild-type enzyme, as it lacked only the first 19 residues. This short region was predicted to be disordered by Phyre2 program [27], and could be unfavorable for protein expression. The two truncated versions of Dga1p were expressed in *E*. *coli* as N-terminus-MBP-fusion proteins. From here and below, Dga1pΔ19 stands for the MBP-Dga1pΔ19 fusion protein, and Dga1pΔ85 for the MBP-Dga1pΔ85 construct. Bacteria expressing pMalc2x with either Dga1pΔ19 or Dga1pΔ85 were disrupted and total extracts were separated into soluble and insoluble fractions. Major bands corresponding to Dga1pΔ19 and Dga1pΔ85 were observed on Coomassie stained polyacrylamide gels, the sizes of which were in accordance with their calculated molecular weights of about 99 kDa and 90 kDa, respectively (Fig 2A). Significant amounts of the target proteins were in the soluble fraction. Soluble crude extracts were firstly purified using amylose affinity chromatography. The MBP-tagged proteins were eluted, providing solutions in which the target enzyme was by far the major protein (Fig 2B): 2 mg of proteins were routinely obtained for one liter of *E*. *coli* culture. Further purification by size exclusion chromatography resulted in highly purified recombinant proteins. The major peaks containing the prominent proteins observed on the chromatograms (Fig 2C) correspond to a dimeric form of the Dga1p constructs. PAGE indicates a good purity of the corresponding fractions. In particular, Dga1pΔ19 has been purified to homogeneity.

### Both recombinant Dga1p constructs synthesize TG *in vitro*

**Fig 3:**
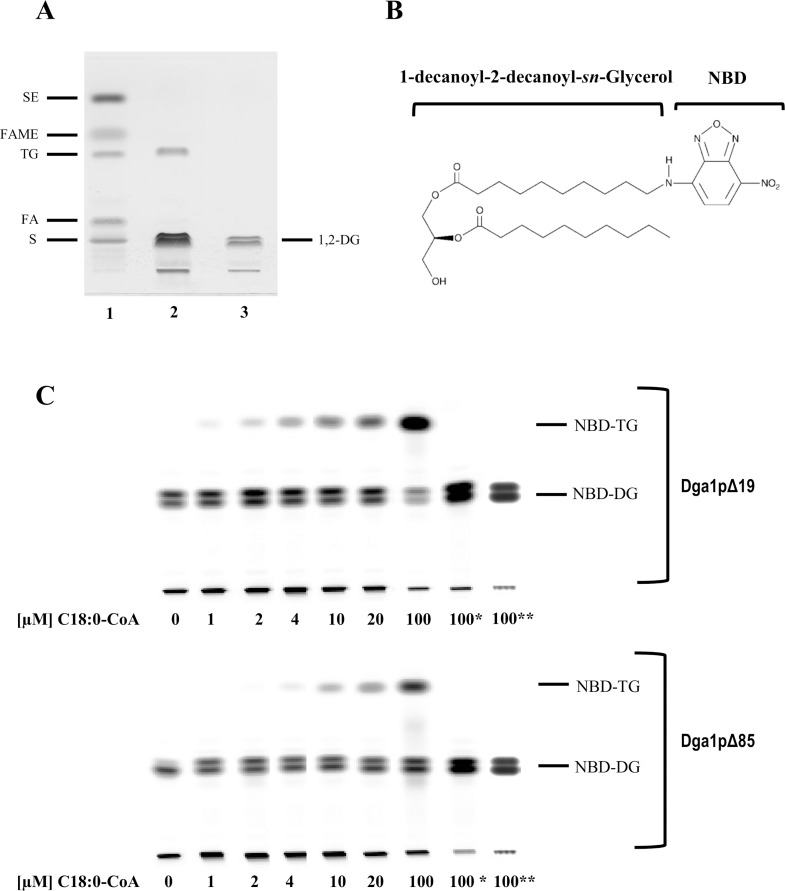
TG production using purified recombinant enzymes. A: HPTLC plate showing the result of DGAT assay using an unlabeled DG acceptor (1,2-Dioleoyl-*sn*-glycerol) and the C18:0-CoA acyl donor, in the presence (lane 2) or the absence (lane 3) of purified Dga1pΔ19. 1,2-DG stands for 1,2-dioleoyl-*sn*-glycerol. Lane 1 contains selected standard lipids: SE: sterol ester, FAME: fatty acid methyl ester; TG: triglyceride; FA: fatty acid; S: sterol. B: Chemical representation of the NBD-DG substrate used in fluorescent DGAT assays. C: HPTLC plates showing dose-response activity of both enzymes using increased concentrations of C18:0 acyl-CoA donor substrate and NBD-DG as acceptor. 100* indicates the control using 100°C boiled enzymes, and 100** the control showing purified MBP protein alone.

To determine whether Dga1pΔ19 and Dga1pΔ85 constructs encode biologically active DGAT2 enzymes, we firstly performed a direct assay using non-labeled C18:0-CoA and 1,2-dioleoyl-*sn-*glycerol substrates. Lipids were separated using automated HPTLC. The purified tagged Dga1pΔ19 produced sufficient TG for detection using copper sulfate charring and staining (Fig 3A). However, a similar assay using the purified Dga1pΔ85 did not result in visible TG spot (data not shown). To optimize the detection of TG, and to perform quantitative measurements, we implemented an assay that uses a fluorescent DG substrate [31]. In this case, the authors used microsomal fractions including total membranes proteins, while we worked with purified proteins, making tests more specific of the enzyme. In the case of the first characterized DGAT2, that of the oleaginous fungus *Mortierella ramanniana*, a preference for C6:0 to C:10 DG acyl acceptors was shown [33]. We therefore used a C:10-C:10 substrate, 1-NBD-decanoyl-2-decanoyl-sn-Glycerol (NBD-DG, Fig 3B), in order to compare the DGAT activity of the two recombinant forms. We verified that an alternate (C:18-C:20) fluorescent DG substrate, 1-NBD-stearoyl-2-arachidonoyl-*sn*-Glycerol, gave rise to NBD-TG production with comparable acyl-CoA substrate specificity (data not shown). Using NBD-DG, the detection limit for lipids was 5.5 ng per band in the fluorescent assay, as compared to 160 ng using conventional lipid staining. With this fluorescent substrate, both recombinant enzymes harbored increasing DGAT activities in the presence of various C18:0-CoA concentrations (Fig 3C). The more active Dga1pΔ19 exhibited a significant consumption of the NBD-DG substrate. Quantification of TG produced showed that, using 100 μM of C18:0-CoA as acyl donor, Dga1pΔ19 produced 3194 pmol of NBD-TG/min/mg of proteins, whereas Dga1pΔ85 produced 1344 pmol of NBD-TG/min/mg of proteins. Thus, Dga1pΔ19 activity was ~2.5 fold higher than Dga1pΔ85 one. In both cases the reaction was proportional to the acyl-CoA amount. Control assays consisted in the incubation of the substrates either with 100°C heated enzymes or with MBP expressed from the empty pMalc2X vector; they did not lead to the production of fluorescent TG.

### Substrate specificity of recombinant Dga1p enzymes

**Fig 4:**
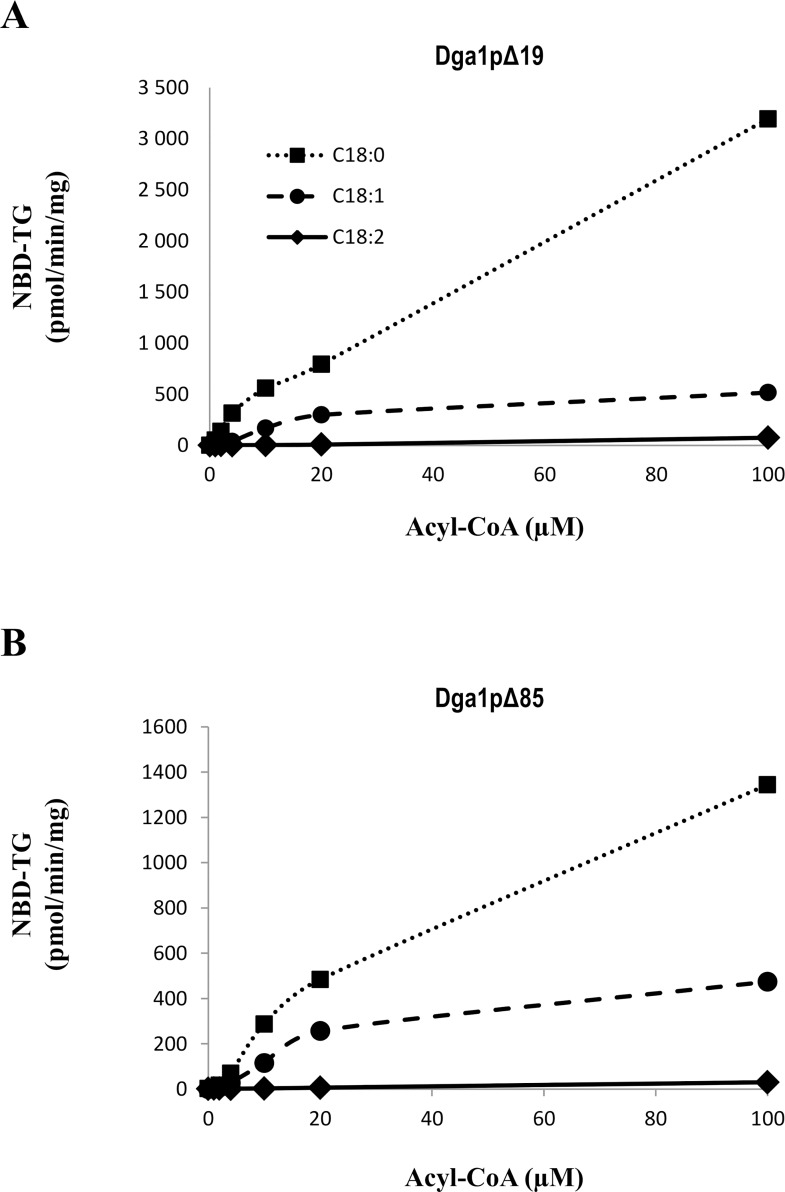
Both Dga1p constructs prefer a saturated acyl-CoA substrate. Dose-response activity of Dga1pΔ19 (A) and Dga1pΔ85 (B). DGAT activity was assayed on 8 μg of purified proteins in the presence of 100 μM NBD-DG, and using increasing concentrations of acyl-CoA substrates (0–100 μM). Acyl donors used were C18:0-CoA (coefficient of determination R^2^ = 0.9966 for Dga1pΔ19 and R^2^ = 0.9981 for Dga1pΔ85), C18:1-CoA (R^2^ = 0.9963 for Dga1pΔ19 and R^2^ = 0.9959 for Dga1pΔ85) and C18:2-CoA (R^2^ = 0.9992 for both Dga1pΔ19 and Dga1pΔ85).

To gain further insight into the specificity of the two produced versions of Dga1p enzymes, we assayed different acyl-CoA substrates. In the yeast *Y*. *lipolytica*, the most abundant fatty acids are oleic acid (C18:1) and linoleic acid (C18:2), their relative proportions depending on culture conditions [34]. In addition, C18:1-CoA is the preferred substrate for Dga1p enzyme of the yeast *S*. *cerevisiae* [35]. Taking into account these published data, we firstly assayed three C18-CoA substrates differing by the number of unsaturations, namely C18:0-CoA, C18:1-CoA and C18:2-CoA (Fig 4). C18:0-CoA was an efficient acyl donor for both enzyme versions. DGAT activities measured in the presence of unsaturated acyl-CoAs were lower, as indicated by the production of 76, 517 and 3194 pmol NBD-TG/min/mg of proteins in the presence of C18:2-CoA, C18:1-CoA or C18:0-CoA, respectively (Fig 4A; activities calculated in the presence of 100 μM acyl-CoA). A similar trend was obtained for Dga1pΔ85, resulting in the production of 30, 474 and 1344 pmol NBD-TG/min/mg of proteins (Fig 4B).

**Fig 5:**
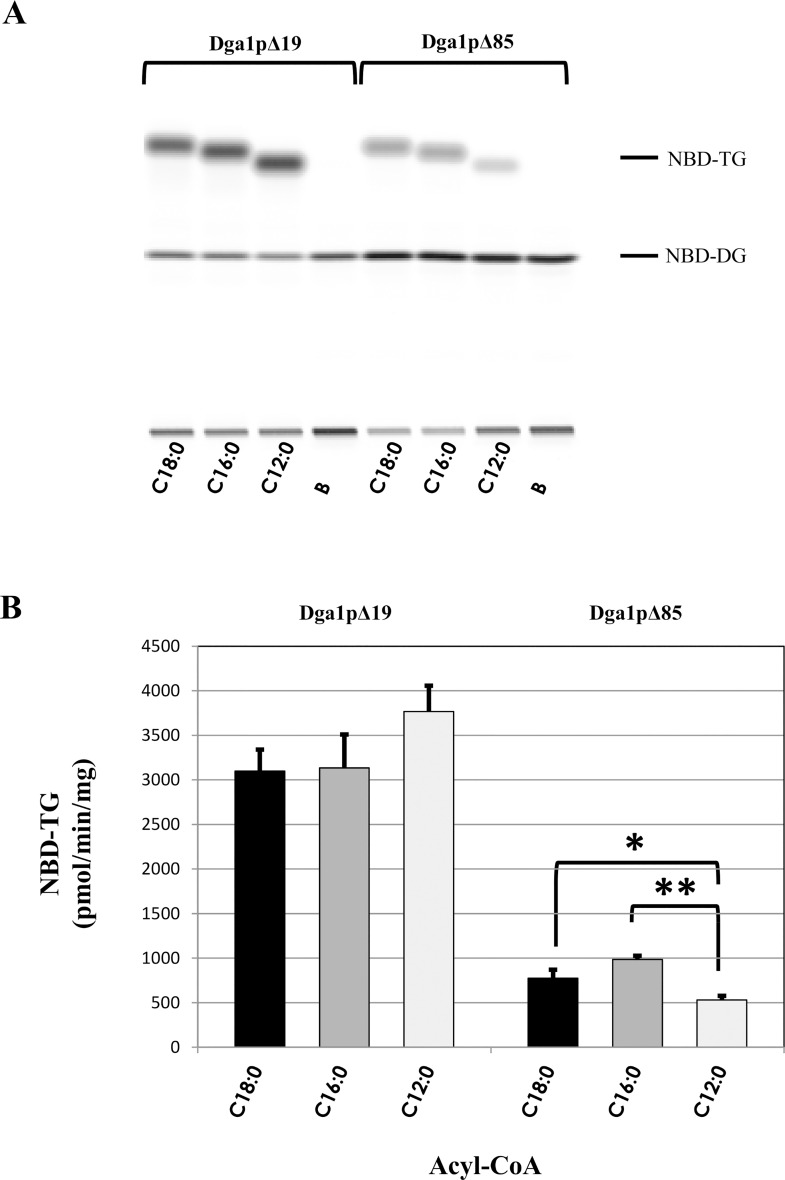
Dga1p enzymes can integrate saturated substrates of different length. A: HPTLC plate showing the production of NBD-TG that integrated acyl-CoA donors of different length. B stands for the control in the presence of boiled enzyme. B: Quantification of NBD-TG produced either by Dga1pΔ19 or by Dga1pΔ85 in the presence of 50 μM of acyl-CoA substrate. Results are expressed as the mean values of triplicates ± standard deviations (error bars). Asterisks indicate statistically significant differences according to a Student’s *t*-test (*, p<0.05; **: p<0.001).

In a second approach, we assayed acyl-CoA substrates of different chain lengths. As C18:0-CoA was the best substrate for both recombinant enzymes, we compared it with other saturated substrates: we assayed C16:0-CoA and C12:0-CoA, in the same conditions as described above. All substrates were efficiently integrated by the two enzyme versions (Fig 5). In the case of Dga1pΔ85, the C12:0-CoA substrate was less efficiently used than its longer counterparts.

### Niacin negatively affects Dga1p activity

**Fig 6:**
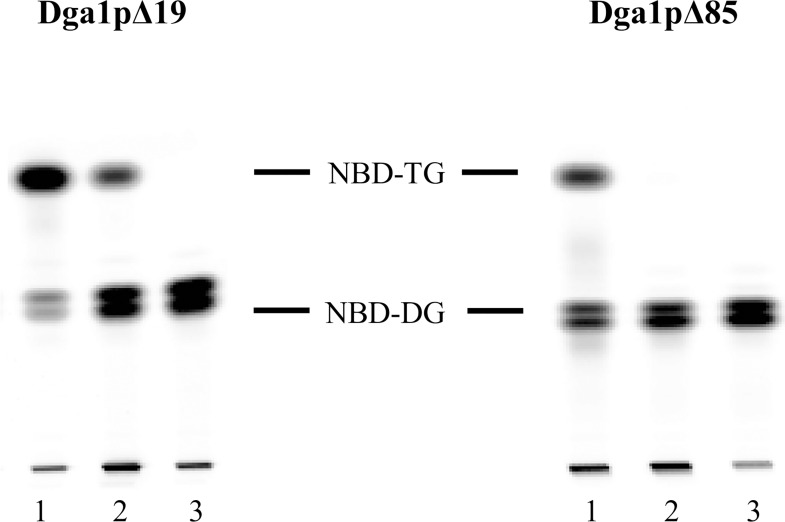
Niacin inhibits Dga1p activity. HPTLC plates showing on lanes 1 the enzyme activity in the presence of 100 μM C18:0-CoA and 100 μM NBD-DG, on lanes 2 the activity when adding 1 mM niacin, on lanes 3 the control in the presence of boiled enzyme.

To confirm that both Dga1p enzyme versions exhibited DGAT2 specific activity, we assayed them in the presence of 1mM niacin, which has been described as a specific noncompetitive DGAT2 inhibitor [36]. In the assay reaction mixture containing 100 μM of C18:0-CoA and NBD-DG as substrates, HPTLC results indicated that the presence of niacin significantly decreased the production of TG (Fig 6). This decrease was 70% for Dga1pΔ19 and up to 99.5% for Dga1pΔ85.

## Discussion

Fully elucidating the molecular mechanisms underpinning DGAT-mediated catalysis is crucial for the development of novel biotechnological strategies aimed at tailoring TG synthesis. However, due to the difficulty to purify DGAT enzymes to homogeneity, little is known about these mechanisms. In this work, we focused on the functional characterization of the *Yarrowia lipolytica* DGAT2 enzyme, encoded by the *DGA1* gene, through the production of the protein in *E*. *coli*. *DGA1* has been shown to be important in lipid accumulation in both *Y*. *lipolytica* [17] and *S*. *cerevisiae* [19].

### Dga1p is expressed in *E*. *coli* as soluble and active forms exhibiting DGAT2 features

Several DGAT2 enzymes have been expressed in various hosts including yeasts, plants [30], insects [4] and mammals [20]. Overexpression studies of these proteins were also reported in *E*.*coli*. Tung tree DGAT2 was expressed and purified from *E*.*coli* but was not active [26]. Peanut (*Arachis hypogea*) DGAT2 genes were also overexpressed in *E*. *coli* as GST fusion proteins and the FA composition of transformed bacteria was analyzed [25]. However, the production of a purified and functionally active DGAT2, upon expression in *E*.*coli*, has not been reported so far. This hampers detailed structural and *in vitro* enzymatic studies, thus limiting the possibility to engineer DGAT2 enzymes. In addition, purification of DGAT proteins from any source is particularly difficult, due in part to the presence of several TM domains [26]. Moreover, DGAT2 enzymes can be very unstable proteins rapidly degraded by the proteasome, as recently shown for murine DGAT2 [37], rendering their study even more difficult. Here, we successfully expressed in *E*.*coli* and purified two functional forms of Dga1p fused at the amino-terminus to MBP, which is known to increase the solubility of target proteins [38]. We engineered two recombinant forms: Dga1pΔ19 and Dga1pΔ85. We demonstrated their activities, using a DGAT fluorescent assay modified from [31].

**Fig 7:**
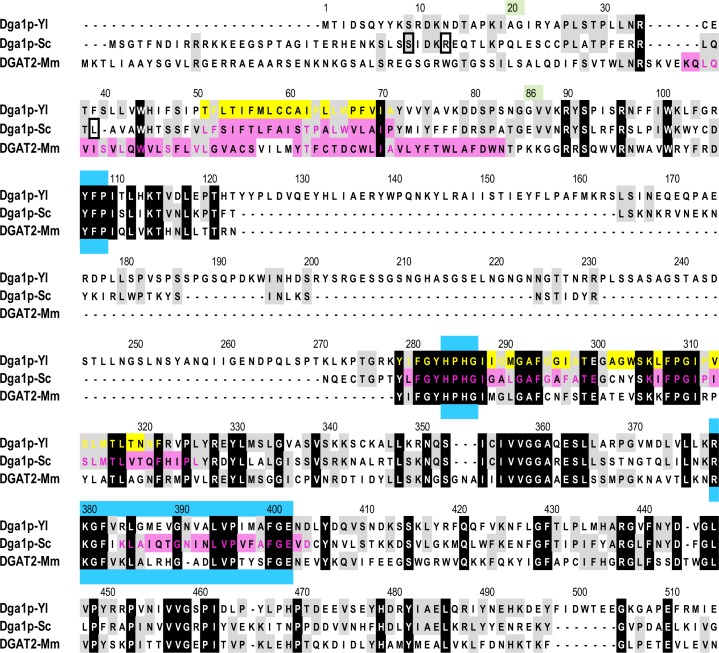
Sequence alignment of DGAT2 enzymes. Dga1p-Yl: enzyme from *Yarrowia lipolytica*, Dga1p-Sc: *Saccharomyces cerevisiae*, DGAT2-Mm: *Mus musculus*. Alignment was performed using ClustalW [47]. Residues conserved in all three enzymes are on a black background, those conserved in two enzymes are on a grey background. TM domains are indicated in pink, as experimentally determined for Dga1p-Sc [39] and DGAT2-Mm [41]. TM domains predicted for Dga1p-Yl are yellow. The green background indicates the N-terminal residue fused to MBP in the recombinant soluble forms of Dga1p-Yl. The three regions highly conserved in DGAT2 enzymes and important for activity are emphasized up and down with blue rectangles.

As mentioned above, we could not express the full-length Dga1p enzyme, the closest construct of which we used being Dga1pΔ19. A possible contribution to enzyme activity of the short 19-residues N-terminal sequence is not supported by the published literature for the model yeast *S*. *cerevisiae* Dga1p. Whereas the mutant lacking the entire hydrophilic N-terminus (Δ1–62; truncation positions 33, 37 and 62 mentioned here are boxed on Fig 7) presented only minimal activity, removal of the first 33 residues resulted in a very minor activity decrease [39]. Interestingly, in the *Δsnf2* disruptant (defective for a DNA-dependent ATPase) of *S*. *cerevisiae*, which has a higher lipid content than the wild-type yeast, overexpression of the N-terminal truncated Dga1p (Δ1–37 mutant) resulted in an increased activity of the lipid body fraction, as compared with overexpression of the full-length Dga1p [40].

Addition of niacin, described to specifically target DGAT2 enzymes, inhibited the activity of both constructs. The inhibition, nearly complete with Dga1pΔ85, was significantly lower with Dga1pΔ19. A possible explanation is that the predicted N-terminal TM domain of Dga1p helps overcoming the inhibitory effect.

### The putative N-terminal transmembrane domain of Dga1p appears important, but not necessary for enzyme activity

The NBD-TG formation we observed was higher using Dga1pΔ19 than Dga1pΔ85. The latter construct still retains a significant DGAT activity, suggesting that residues 51–71, predicted to constitute a TM domain of Dga1p (yellow in Fig 7), participate to enzyme activity, but are not necessary for it. These results are in contrast with previous work based on mutagenesis analysis of *S*. *cerevisiae* Dga1p [39]. In this study, the deletion of the first N-terminal TM domain (Δ70–91 mutant) resulted in a total loss of activity. Our results are closer to those obtained with murine DGAT2, for which the deletion variant lacking the first TM domain (Δ66–115) still retained catalytic activity [23], even though this domain contains the consensus sequence (FL*X*L*XXX*^*n*^, where ^n^ is a nonpolar *amino* acid) for a putative neutral lipid-binding domain. This lipid-binding domain is not conserved in yeast DGAT2s. The observed activity of Dga1pΔ85 is in accordance with the absence of a conserved motif in the deleted N-terminal fragment. Indeed, six regions conserved in DGAT2 have been identified [21]. Among them, three motifs (emphasized in blue in Fig 7) were shown to be important for activity, using substitution or chemical modification. Firstly, all DGAT2 members, from animals to fungi, contain the conserved sequence HPHG (EPHS in plants). This HPHG motif, part of a TM domain in *S*. *cerevisiae* Dga1p, is important for its catalytic function [39]. HPHG is also essential for murine DGAT2 activity [41], in which it is outside TM domains. Secondly, another conserved sequence, YFP, significantly contributes to the activity of *S*. *cerevisiae* DGAT2 [39]. Finally, the most conserved motif in DGAT2s, RXGFX(K/R)XAXXXGXX(L/V)VPXXXFG(E/Q)) should also contribute to enzyme activity [42]. All these conserved motifs are present (the third one in Dga1p is slightly modified to RXGFXRXGXXXGXXXLVPXXXFGE) in our constructs.

### Dga1p prefers long chain saturated acyl-CoAs as acyl donors *in vitro*

We also analyzed the substrate preference of the recombinant Dga1p, which showed that saturated substrates (C18:0-CoA, C16:0-CoA and C12:0-CoA) were efficiently used, whereas C18:1-CoA substrate integration was less than half as efficient. Moreover, the observed product level with linoleoyl-CoA (C18:2-CoA) was not significant. We hypothesize that the presence of double bonds in the acyl chains imposes a more constrained structure which is not in favor of good substrate utilization, as compared to the more flexible saturated ones. The preference of recombinant Dga1p constructs for saturated substrates differs from several results obtained for other DGAT2 species. For instance, mouse DGAT2 enzyme expressed in insect cells can integrate FA-CoAs with various degrees of unsaturation, preferring C18:1-CoA followed by C16:0-CoA [5]. *Arabidopsis thaliana* DGAT2 has a relative preference for C18:2-CoA or C18:1-CoA [43]. In the plant enzymes from castor bean [6], tung tree [26], and *Arabidopsis thaliana* [44], DGAT2 is specific for unsaturated FA. *S*. *cerevisiae* Dga1p preferentially incorporates C18:1-CoA and C16:0-CoA, the activity observed for both substrates being twice that for C18:0-CoA [35]. These differences in specificity could be attributable: i) to the fact that all assays in these works were done on membrane or microsomal extracts, while we used purified enzymes. DGAT catalysis may involve molecular partners from the host that are absent in the *in vitro* experiments. ii) to differences between species, iii) to the different systems used for heterologous expression. In addition, our *in vitro* results were performed in the presence of tween detergent, thus suppressing the solubility differences between substrates that could be important *in vivo*. The above mentioned points could also explain why Dga1p prefers saturated substrates *in vitro* whereas the most abundant fatty acids in *Y*. *lipolytica* are unsaturated ones.

## Conclusions

This work describes the efficient production of functional DGAT enzymes, using two recombinant Dga1p proteins expressed in *E*. *coli* as MBP fusion proteins. Our results indicate that both purified Dga1pΔ19 and Dga1pΔ85 enzyme versions efficiently catalyze the esterification of acyl-CoA donors, containing various FAs, with different DG acceptors. Differences in activity between the two enzyme versions may be due to the presence of a TM domain. We also observed that the two recombinant enzymes preferentially incorporated saturated acyl-CoA substrates. These DGAT2 activities were validated by adding 1mM of niacin, a DGAT2 specific inhibitor. To our knowledge, for the integral membrane proteins DGATs, this is an unprecedented example of production of a soluble and active enzyme version purified in large amounts. The two Dga1p constructs produced here represent valuable models for functional and structural studies on DGAT2 family. Such structural work could take advantage of various biophysical techniques, for instance circular dichroism, small-angle X-ray scattering, protein crystallography or cryo-electron microscopy. A deeper structure-function knowledge should help engineering DGAT2 enzymes in order to produce yeast or plant strains synthesizing medium-chain fatty acids for biofuels, or unusual fatty acids, such as polyunsaturated fatty acids [45] or hydroxy fatty acids [6], for green chemistry.
